# A Hybrid Deep Learning and Machine Learning Approach with Mobile-EfficientNet and Grey Wolf Optimizer for Lung and Colon Cancer Histopathology Classification

**DOI:** 10.3390/cancers16223791

**Published:** 2024-11-11

**Authors:** Raquel Ochoa-Ornelas, Alberto Gudiño-Ochoa, Julio Alberto García-Rodríguez

**Affiliations:** 1 Systems and Computation Department, Tecnológico Nacional de México/Instituto Tecnológico de Ciudad Guzmán, Ciudad Guzmán 49100, Mexico; 2 Electronics Department, Tecnológico Nacional de México/Instituto Tecnológico de Ciudad Guzmán, Ciudad Guzmán 49100, Mexico; m21290934@cdguzman.tecnm.mx; 3 Centro Universitario del Sur, Departamento de Ciencias Computacionales e Innovación Tecnológica, Universidad de Guadalajara, Ciudad Guzmán 49000, Mexico; julio.garciar@cusur.udg.mx

**Keywords:** lung cancer, colon cancer, artificial intelligence, histopathological images, machine learning, deep learning, MobileNet, EfficientNet

## Abstract

Background: Lung and colon cancers are among the most prevalent and lethal malignancies worldwide, underscoring the urgent need for advanced diagnostic methodologies. This study aims to develop a hybrid deep learning and machine learning framework for the classification of Colon Adenocarcinoma, Colon Benign Tissue, Lung Adenocarcinoma, Lung Benign Tissue, and Lung Squamous Cell Carcinoma from histopathological images. Methods: Current approaches primarily rely on the LC25000 dataset, which, due to image augmentation, lacks the generalizability required for real-time clinical applications. To address this, Contrast Limited Adaptive Histogram Equalization (CLAHE) was applied to enhance image quality, and 1000 new images from the National Cancer Institute GDC Data Portal were introduced into the Colon Adenocarcinoma, Lung Adenocarcinoma, and Lung Squamous Cell Carcinoma classes, replacing augmented images to increase dataset diversity. A hybrid feature extraction model combining MobileNetV2 and EfficientNetB3 was optimized using the Grey Wolf Optimizer (GWO), resulting in the Lung and Colon histopathological classification technique (MEGWO-LCCHC). Cross-validation and hyperparameter tuning with Optuna were performed on various machine learning models, including XGBoost, LightGBM, and CatBoost. Results: The MEGWO-LCCHC technique achieved high classification accuracy, with the lightweight DNN model reaching 94.8%, LightGBM at 93.9%, XGBoost at 93.5%, and CatBoost at 93.3% on the test set. Conclusions: The findings suggest that our approach enhances classification performance and offers improved generalizability for real-world clinical applications. The proposed MEGWO-LCCHC framework shows promise as a robust tool in cancer diagnostics, advancing the application of AI in oncology.

## Simple Summary

Lung and colon cancers are among the leading causes of death globally. Accurate and early detection is essential for improving patient outcomes. However, the existing dataset used in many studies for cancer diagnosis is limited by augmentation methods, reducing its real-world applicability. This study addresses the limitations by introducing new real-world histopathological images to enhance the dataset and applying advanced contrast enhancement techniques. Our proposed model integrates feature extraction from MobileNetV2 and EfficientNetB3 with Grey Wolf Optimization for feature selection and classification. This approach improves the model’s generalizability, offering better support for pathologists in real-time cancer diagnosis.

## 1. Introduction

Cancer remains one of the leading causes of mortality worldwide, with lung and colon cancers being among the most prevalent. According to recent statistics from the World Health Organization, lung cancer is responsible for approximately 2.2 million new cases and 1.8 million deaths annually, making it the leading cause of cancer-related deaths globally [1]. Similarly, colon cancer accounts for 1.9 million new cases and over 900,000 deaths each year [2]. Notably, nearly 17% of patients with lung cancer are also diagnosed with colon cancer simultaneously, indicating a significant overlap between these two malignancies [3]. This dual incidence underscores the need for precise and efficient diagnostic tools, as the concurrent presence of both cancers complicates clinical management and increases the mortality risk.

Lung cancer severely affects the respiratory system, often presenting with symptoms such as persistent cough, hemoptysis, shortness of breath, and chest pain [4]. In contrast, colon cancer primarily impacts the digestive system, with symptoms including rectal bleeding and abdominal discomfort [5]. While non-invasive methods such as computed tomography (CT) imaging and radiography are widely used for detecting lung and colon cancers, these techniques are not always reliable for definitive diagnosis [6,7]. Invasive procedures, particularly histopathological analysis of tissue samples, remain the gold standard for accurate cancer detection and treatment planning. However, manual examination of histopathological images by pathologists is time-consuming, labor-intensive, and prone to human error, especially in early-stage cancers where visual patterns may be subtle [8,9].

Recent advancements in artificial intelligence (AI) and deep learning (DL) offer promising solutions for automating cancer diagnosis, reducing the burden on pathologists, and improving diagnostic accuracy [10]. DL models, particularly convolutional neural networks (CNNs), have shown remarkable success in detecting complex patterns within histopathological images [11]. These models are capable of extracting features that may not be immediately apparent to the human eye, thereby facilitating the early detection and classification of cancers at various stages. The role of DL extends to several medical imaging modalities, including magnetic resonance imaging (MRI), radiographs, and retinal images, enhancing both diagnostic precision and the speed of decision-making [12,13].

However, developing and implementing efficient algorithms in this domain requires large, well-annotated datasets. This is particularly challenging in medical image analysis, where data scarcity often limits model generalization [14]. Consequently, transfer learning (TL) and deep learning techniques are crucial for building robust diagnostic mechanisms. TL allows pre-trained models, typically trained on large-scale datasets such as ImageNet, to be adapted to medical imaging tasks with relatively smaller datasets, thereby accelerating the development process and enhancing model performance [15,16].

In recent years, models such as MobileNetV2 and EfficientNetB3-B7 have emerged as promising solutions, offering a balance between computational efficiency and classification accuracy. These models have demonstrated superior performance in medical image classification tasks, particularly in the context of histopathological image analysis [17,18,19,20].

Nevertheless, many studies still rely on the LC25000 dataset, which consists of 25,000 images generated through augmentation from only 750 original histopathological images of lung and colon tissues across five classes [21,22,23,24]. Consequently, models trained solely on this dataset may struggle to generalize to real-world clinical settings due to its limited diversity. This highlights the necessity for more robust datasets and advanced feature extraction techniques to enhance the accuracy and reliability of AI-based diagnostic tools [25]. The success of these models often depends on crucial preprocessing steps, such as contrast enhancement and color adjustment, which help reveal subtle features that are essential for accurate diagnosis [24,26,27].

Despite recent advancements in AI and DL for cancer diagnostics, current methodologies still face significant limitations when applied to lung and colon cancer detection. One primary issue is the lack of real-world applicability, as many studies rely heavily on augmented datasets with limited diversity, such as the LC25000 dataset, which may restrict generalization to varied clinical settings [22,23,24,25,26,27]. Additionally, the reliance on complex model architectures often requires specialized hardware, limiting their practicality in resource-constrained environments. Furthermore, the current techniques may struggle with accurate differentiation between similar histopathological features, especially in early-stage cancers where visual cues are subtle. Addressing these limitations is crucial to developing more reliable and efficient diagnostic tools [18,22,23,24,25].

In this manuscript, a novel hybrid approach is proposed, combining MobileNetV2 and EfficientNetB3 for feature extraction with GWO to reduce the feature space. The proposed MEGWO-LCCHC technique is further validated using an enhanced dataset that includes 1000 new histopathological images sourced from the National GDC Cancer Portal, replacing augmented images from the original LC25000 dataset. This robust and generalized dataset, coupled with contrast enhancement using CLAHE, improves the model’s performance. The proposed method is tested using both machine learning and deep learning models, including a deep neural network (DNN), demonstrating its suitability as a lightweight, real-time diagnostic tool for pathologists. The key contributions of this work include:

- The introduction of a hybrid feature extraction approach combining MobileNetV2 and EfficientNet for efficient and accurate cancer detection.
- The application of GWO to reduce the feature space by 38%, improving computational efficiency while maintaining high classification accuracy.
- The integration of new images from the National Cancer Institute GDC Data Portal to enhance the generalizability of the model.
- The demonstration of the model’s performance using both machine learning (ML) and DL models, including a lightweight DNN model.

The rest of the paper is structured as follows: Section 2 provides a comprehensive review of the relevant literature. In Section 3, the proposed model is explained in detail. Section 4 focuses on the evaluation of the model’s performance, and Section 5 offers concluding remarks and potential future directions for this research.

## 2. Related Works

In 2020, Garg et al. [28] explored the utility of pre-trained CNN models for analyzing histopathological images to predict lung and colon cancer. Their findings indicated high accuracy levels, achieving 96.67% for classifying colon tissues and 95.8% for breast cancer detection by harnessing textural and architectural features. They further enhanced the classification performance of colon biopsy images to 98% by integrating a hybrid feature set with a Support Vector Machine (SVM). However, the reliance on feature engineering and the division of datasets into binary categories raised concerns about the generalizability of their results across diverse datasets.

Building on this foundation, Masud et al. [7] advanced the field in 2021 by employing a deeper CNN architecture tailored for classifying lung and colon cancer histopathological images. Their model incorporated three convolutional layers and two max-pooling layers, attaining an accuracy of 88.1% along with precision and sensitivity values of 84.6% and 91.3%, respectively. Despite this robust framework, the model’s performance suggests limitations in generalizing across varied datasets, especially when faced with complex histopathological characteristics.

In the same year, Ali et al. [29] introduced a novel dual-stream capsule network for the classification of lung and colon cancers. This innovative architecture, which integrated conventional and separable convolutional layers, recorded an accuracy of 98.66% and a sensitivity of 99.06%. Nonetheless, the complexity of capsule networks may present challenges for practical implementation, particularly in resource-limited clinical settings.

As research progressed, 2022 saw the introduction of more sophisticated techniques. Talukder et al. [30] proposed an ensemble learning method combined with deep feature extraction, achieving accuracy levels exceeding 99% for lung and colon cancer detection. Their approach utilized k-fold cross-validation and the LC25000 dataset for feature extraction, providing a comprehensive evaluation framework. However, the computational demands associated with ensemble methods may restrict their practical application in real-time clinical diagnostics.

Another significant contribution that year came from Hage et al. [31], who employed feature engineering to extract 37 distinct features, including first-order statistics and Gray Level Co-Occurrence Matrix (GLCM), for lung and colon cancer classification. Their optimization of parameters through Unsharp Masking resulted in a classification accuracy of 95.6%. This study emphasizes the critical importance of feature selection while also highlighting the challenges of manual feature selection, which may overlook subtle nuances in histopathological images.

The exploration of lightweight deep learning models gained traction in 2022, as demonstrated by Attallah et al. [22], who achieved an impressive 99.6% accuracy in lung and colon cancer classification using ShuffleNet, MobileNet, and SqueezeNet models. Their methodology included principal component analysis (PCA) and fast Walsh–Hadamard transform (FHWT) for feature reduction, employing classifiers such as Linear Discriminant Analysis (LDA), Quadratic Discriminant Analysis (QDA), SVM, and ESD. Similarly, Yahia Ibrahim et al. [27] focused on enhancing diagnostic accuracy through advanced preprocessing techniques, utilizing Double CLAHE in conjunction with deep learning strategies. Their CNN model, fine-tuned with EfficientNetB7 pre-trained weights, achieved an impressive accuracy of 99.5% and a precision of 99.6%.

The year 2023 heralded significant advancements in optimizing deep learning models. Mengash et al. [24] combined the Marine Predators Algorithm (MPA) with deep learning, resulting in the MPADL-LC3 model, which achieved a classification accuracy of 99.27%. This integration illustrates the potential of nature-inspired algorithms to improve diagnostic performance, though the complexity of implementation in clinical practice remains a challenge. In a related study, Al-Jabbar et al. [26] proposed a hybrid approach that combined hand-crafted features with those extracted by CNNs, achieving classification accuracies ranging from 99% to 100% on the LC25000 dataset using CLAHE and PCA for feature enhancement. While this approach underscores the value of feature fusion, reliance on manual extraction may limit scalability and introduce variability across datasets.

Further advancements were made by AlGhamdi et al. [23] in 2023, leveraging the BERTL-HIALCC method to achieve precision levels above 98% for specific cancer classes. Their model demonstrated robustness through advanced feature extraction and parameter optimization, although the complexity of the approach—incorporating algorithms like ShuffleNet and DCRNN—may require substantial computational resources, posing barriers to widespread clinical application. Additionally, Alqahtani et al. [18] developed an enhanced Water Strider algorithm integrated with a Convolutional Autoencoder, achieving 99.41% accuracy and notable computational efficiency. This innovative approach highlights the potential of merging advanced optimization algorithms with deep learning to elevate diagnostic accuracy, though practical deployment necessitates further exploration.

Obayya et al. [32] implemented the BICLCD-TSADL model, combining the Tuna Swarm Algorithm with deep learning techniques to achieve exceptional precision in detecting lung and colon cancers, surpassing 97% in precision, recall, F-score, and AUC-score for both cancer types. This study demonstrates the promise of hybrid algorithms in enhancing diagnostic accuracy, although the computational demands may challenge scalability. Tummala et al. [33] introduced an explainable deep learning model for classifying lung and colon carcinoma, attaining 99.96% accuracy on the LC25000 dataset. However, reliance on advanced techniques may necessitate further validation across diverse clinical datasets to ensure robustness and generalizability.

In 2024, Hasib Uddin et al. [34] proposed a multi-modal approach for classifying colon and lung cancers using advanced neural network architectures. Their model achieved an impressive classification accuracy of 98.4%, significantly enhancing diagnostic performance compared to existing methods. This study emphasized the integration of histopathological and radiological images, which improved feature extraction and resilience against data variability. Lastly, Hasan et al. [35] proposed a multi-scale CNN integrated with an explainable AI (XAI) system for lung and colon cancer classification, achieving an accuracy of 98.40%. The incorporation of XAI techniques enhances transparency in model decision-making, addressing a critical need for interpretability in AI-driven diagnostics. Nonetheless, the deployment of such systems in clinical settings requires careful consideration of the balance between model complexity and practical applicability.

The selection of MobileNetV2 and EfficientNetB3 for our proposed MEGWO-LCCHC method is motivated by their proven effectiveness and superior performance metrics in the classification of histopathological images. By integrating these advanced architectures, we aim to create a model that not only enhances classification accuracy but also emphasizes computational efficiency. This approach addresses a critical gap in the current literature, where many studies primarily rely on the widely used LC25000 dataset, potentially limiting the generalizability of their findings across diverse populations and clinical settings. Our strategy seeks to enrich the dataset with a more diverse set of images while ensuring that the resulting model remains lightweight and suitable for real-time applications.

## 3. Proposed Model

**Figure 1:**
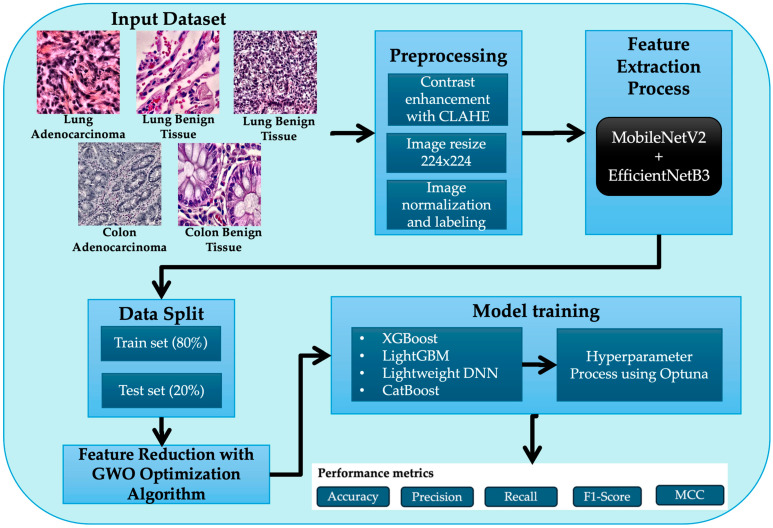
Overview of the proposed MEGWO-LCCHC model for colon and lung cancer detection.

In this manuscript, we present a novel technique for the detection of colon and lung cancer, termed the MEGWO-LCCHC method. The primary objective is to effectively identify colon and lung cancer in both malignant and benign tissues, leveraging a robust feature extraction and classification model. To achieve this, our approach follows a series of subprocesses, which include contrast enhancement of histopathological images using CLAHE, a hybrid feature extraction model based on MobileNetV2 and EfficientNetB3, feature reduction utilizing the GWO, and classification through gradient boosting models (XGBoost, LightGBM, and CatBoost). Additionally, we employ a lightweight DNN for comparative analysis, supported by Optuna-based hyperparameter optimization and cross-validation. Figure 1 illustrates the overall workflow of the proposed MEGWO-LCCHC model and its classification process.

The images used in this study were originally obtained from the LC25000 dataset, which consists of 25,000 histopathological images distributed across five classes: lung squamous cell carcinomas, colon adenocarcinomas, lung adenocarcinomas, and benign lung and colonic tissues [21].

Each class contains 5000 images, totaling 25,000, which were initially augmented from 750 original images that have been validated and widely used in AI application studies. To enhance robustness and generalizability—especially in validating different population groups within clinical applications—the original dataset was enriched by replacing 1000 images of colon adenocarcinoma, lung adenocarcinoma, and lung squamous cell carcinoma sourced from the National Cancer Institute GDC Data Portal, as these three classes represent the most prevalent cancers in the original dataset. The selection of these new images was based on primary diagnosis criteria for adenocarcinoma tumors, focusing on the most relevant cases.

Image extraction was conducted using Aperio ImageScope, a tool commonly employed by pathologists [36]. The extraction process involved applying magnifications ranging from 20× to 40× on regions where the primary diagnosis was established. The dataset utilized in this study is summarized in Table 1.

### 3.1. Contrast Enhancement with CLAHE

CLAHE was applied to enhance the contrast of histopathological images. This method improves local contrast and adapts to the intensity distribution in different regions of the image, making it particularly suitable for medical imaging tasks where details such as tissue structures are crucial [37]. CLAHE works by dividing the input image into non-overlapping contextual regions and applying histogram equalization within each region. This prevents over-amplification of noise in homogeneous regions of the image.

For this process, the parameters were set as follows: a clip limit of 3.0 to control contrast amplification and a grid size to define the size of the contextual regions. This configuration ensured optimal contrast enhancement, avoiding over-saturation of pixels in high-intensity areas while preserving important tissue features.

**Figure 2:**
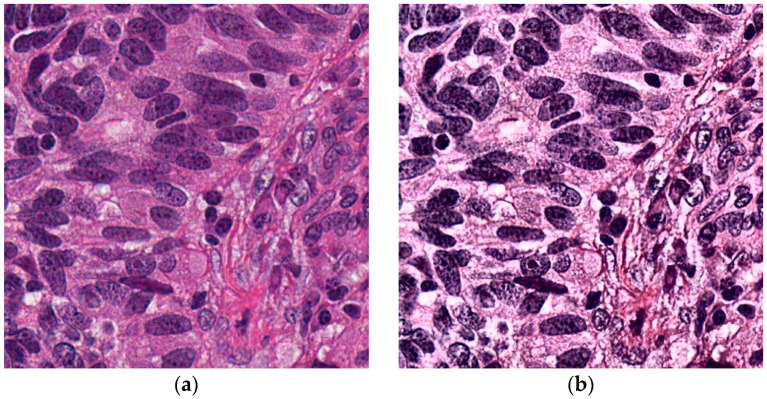
Contrast enhancement of histopathological images using CLAHE: (**a**) Original image of lung adenocarcinoma tissue; (**b**) Enhanced image demonstrating improved local contrast and detail in tissue structures.

By enhancing the contrast in this manner, the model can more effectively distinguish between different tissue types, thereby improving the feature extraction process in subsequent steps [24,26,27]. This not only assures the quality of images in each dataset but also facilitates the appropriate extraction of features following the application of the GWO. Figure 2 illustrates the contrast enhancement achieved under these conditions using images sourced from the National Cancer Institute GDC Data Portal for lung adenocarcinoma tissue.

### 3.2. Feature Extraction Using Hybrid Model with MobileNetV2 and EfficientNetB3

The feature extraction process employed in this study leverages a hybrid model that integrates MobileNetV2 and EfficientNetB3, two efficient and lightweight CNNs [17,18,19,20]. These models were selected based on their balance of accuracy and computational efficiency, making them ideal for real-time classification in histopathology.

The goal is to utilize the strengths of both models to capture high-level abstract features while maintaining computational feasibility. All input images were resized to a fixed dimension of 224×224×3 to ensure compatibility with both CNNs and before being passed to the feature extraction networks. The preprocessing involves image normalization where each pixel value $x_{i,j,k}$ is scaled within the range 0, 1 by:

(1) $$x_{i,j,k}^{\prime }=\frac{x_{i,j,k}}{255}$$

where i, j, k represent the pixel coordinates and color channels, respectively. This section may be divided by subheadings. It should provide a concise and precise description of the experimental results, their interpretation, as well as the experimental conclusions that can be drawn. Let the input image be represented as:

(2) $$I_{rgb}\in R^{224\times 224\times 3}$$

where $I_{rgb}$ denotes the resized RGB image. The feature extraction process for MobileNetV2 can be expressed as follows:

(3) $$F_{MobileNetV2}=MaxPool\left(f_{MobileNetV2}\left(I_{rgb}\right)\right)$$

where f means flatten to obtain a feature vector and $f_{MobileNetV2}$: $R^{224\times 224\times 3}\to R^{h\times w\times c}$ is the convolutional feature mapping function, and the Max Pooling operation reduces the spatial dimensions by applying global max pooling over the feature maps, resulting in $F_{MobileNetV2}\in R^{m}$, where m is the number of features extracted from MobileNetV2 after pooling. Similarly, for EfficientNetB3:

(4) $$F_{EfficientNetB3}=MaxPool\left(f_{EfficientNetB3}\left(I_{rgb}\right)\right)$$

where $f_{EfficientNetB3}$: $R^{224\times 224\times 3}\to R^{h^{\prime }\times {\;w}^{\prime }\times c^{\prime }}$ performs feature extraction, followed by global max pooling to produce $F_{EfficientNetB3}\in R^{n}$, where n is the number of features extracted from EfficientNetB3 after pooling.

The extracted feature vectors $F_{MobileNetV2}$ and $F_{EfficientNetB3}$ are concatenated to form a hybrid feature vector $F_{Hybrid}$, which can be expressed as:

(5) $$F_{Hybrid}=\left[F_{MobileNetV2}∥F_{EfficientNetB3}\right]$$

where $F_{Hybrid}\in R^{m+n}$ represents the combined feature set. representing the combined feature set from both models.

The concatenation of features from both CNNs leverages their complementary strengths. MobileNetV2 excels at capturing detailed low-level features, such as edges, textures, and fine patterns, which are essential for identifying specific structures in histopathological images [17,18,19] These fine details are crucial for recognizing smaller yet significant elements within tissue samples. In contrast, EfficientNetB3 captures high-level semantic features, representing more abstract, complex patterns and relationships, such as overall tissue architecture and tumor presence [20,33]. The fusion of low- and high-level features provides a comprehensive and enriched representation that is vital for accurate classification in medical image analysis.

Both models are initialized with pre-trained weights from the ImageNet dataset, and all layers are frozen during the feature extraction phase to preserve the learned representations [15]. MobileNetV2’s use of inverted residuals and linear bottleneck layers enables efficient capture of low-level details, while EfficientNetB3 balances width, depth, and resolution, effectively capturing high-level semantic features. This hybrid design not only reduces the parameter count, minimizing computational complexity, but also ensures high classification accuracy. This balance between efficiency and accuracy is crucial for real-time diagnostic applications in medical settings, where swift and reliable decision-making is essential. The lightweight nature of the model, with fewer than 13 million parameters, makes it highly suitable for deployment in environments with constrained computational resources, such as point-of-care devices or embedded systems utilized by pathologists.

### 3.3. Grey Wolf Optimizer for Feature Selection Reduction

After extracting the hybrid feature vector $F_{Hybrid}$, a feature selection process was conducted using the GWO. GWO is an optimization algorithm inspired by the social hierarchy and collaborative hunting strategies of grey wolves. By mimicking this natural behavior, the algorithm is well-suited for solving complex optimization problems, such as selecting the most relevant features from high-dimensional data [38,39]. The primary goal of this process is to reduce the dimensionality of the hybrid feature vector while preserving the most informative features, thereby enhancing classification performance without sacrificing accuracy.

Let $F_{Hybrid}\in R^{d}$ represent the concatenated feature vector where d=m+n is the total number of features. The GWO aims to identify the optimal binary mask $b\in {\left\{0,1\right\}}^{d}$, where each element $b_{i}$ indicates whether the i-th feature is selected $\left(b_{i}=1\right)$ or discarded $\left(b_{i}=0\right)$. The reduced feature vector $F_{Reduced}$ is obtained by applying this binary mask:

(6) $$F_{Reduced}=F_{Hybrid}⨀b$$

where ⨀ denotes element-wise multiplication. The optimization seeks to minimize the feature set while maintaining high classification accuracy. The objective function $Fitness\left(b\right)$ for the GWO is defined as:

(7) $$Fitness\left(b\right)=\alpha \times Accuracy-\beta \times \frac{Number\;of\;Selected\;Features}{Total\;Features}$$

where α and β are weight factors that balance classification accuracy and the degree of feature reduction. The GWO algorithm updates the positions of candidate wolves (solutions) using the following equation:

(8) $$X\left(t+1\right)=\frac{X_{\alpha }+X_{\beta }+X_{\delta }}{3}-A\cdot D$$

Here, $X\left(t+1\right)$ represents the updated position of the grey wolf in the search space at iteration t, while $X_{\alpha }$, $X_{\beta }$ and $X_{\delta }$ correspond to the best, second-best, and third-best solutions at the current iteration. A is a control parameter that governs the convergence speed of the wolves toward the optimal solution, D is a distance vector, which helps guide the wolves in exploring the search space. Together, A and D ensure that the wolves balance exploration and exploitation, allowing for a thorough search of the feature space.

This update continues for k=20 iterations with a population size of 12 grey wolves (agents). After 20 iterations, the optimal binary mask is determined, and the reduced feature set is generated as follows:

(9) $$\left|F_{Reduced}\right|=\sum_{i=1}^{d}b_{i}$$

This feature reduction process results in a 38.06% decrease in the size of the feature set. This reduction significantly lowers the computational cost of training the classification model without compromising accuracy. By retaining only the most relevant features, the model becomes more efficient and faster to execute, making it particularly well-suited for real-time classification tasks in clinical environments where both speed and accuracy are critical.

### 3.4. Classification Using ML Models and Hyperparameter Optimization with Optuna

This section presents the ML models used in this study, specifically XGBoost, LightGBM, and CatBoost, with hyperparameter tuning being an integral component in maximizing model performance. Optuna, an efficient framework for hyperparameter optimization, was employed to explore the hyperparameter space using a combination of Bayesian optimization and random search techniques [40]. The objective function minimized during the hyperparameter tuning process is the validation error, measured using cross-validation, and is defined as:

(10) $$Objective\left(\theta \right)=\left(\frac{1}{K}\right)\sum_{k=1}^{K}Loss\left(\theta ,D_{train}^{k},D_{val}^{k}\right)$$

where K represents the number of folds in cross-validation, $D_{train}^{k}$ and $D_{val}^{k}$ are the training and validation sets for the k-th fold, and the loss function can vary (e.g., log loss, accuracy). The cross-validation ensures robust hyperparameter tuning by evaluating performance across multiple data partitions, reducing the likelihood of overfitting. In this study, a 5-fold cross-validation was employed, splitting the dataset into 5 subsets to evaluate model performance across various data configurations.

#### 3.4.1. XGBoost

XGBoost (eXtreme Gradient Boosting) is a decision-tree-based ensemble machine learning algorithm that uses gradient boosting frameworks [41]. The objective function for XGBoost can be defined as:

(11) $$L\left(\theta \right)=\sum_{i=1}^{n}l\left(\hat{y_{i}},y_{i}\right)+\sum_{k=1}^{K}\Omega \left(f_{k}\right)$$

where $l\left(\hat{y_{i}},y_{i}\right)$ represents the loss function (e.g., log loss for classification), $\Omega \left(f_{k}\right)$ is the regularization term that penalizes the complexity of the model. The key hyperparameters tuned for XGBoost include the learning rate, tree depth, number of estimators, and subsampling ratios. Optuna is used to find the optimal hyperparameters, minimizing the validation loss:

(12) $$\theta^{*}=argmin_{\theta }L\left(\theta \right)$$

#### 3.4.2. LightGBM

LightGBM (Light Gradient Boosting Machine) is a gradient boosting framework that utilizes tree-based learning algorithms optimized for speed and memory efficiency [42]. It adopts leaf-wise growth with depth constraints, allowing for faster computations on large datasets. The objective function for LightGBM is given by:

(13) $$L_{LGBM}\left(\theta \right)=\sum_{i=1}^{n}l\left(\hat{y_{i}},y_{i}\right)+\lambda \sum_{j=1}^{d}\omega_{j}^{2}$$

where $\lambda \sum_{j=1}^{d}\omega_{j}^{2}$ is the regularization term that penalizes the leaf weights $\omega_{j}$. The main hyperparameters optimized using Optuna include the learning rate, the maximum depth of the trees, and the number of leaves. Optuna helps minimize the objective function:

(14) $$\theta^{*}=argmin_{\theta }L_{LGBM}\left(\theta \right)$$

#### 3.4.3. CatBoost

CatBoost (Categorical Boosting) is an efficient gradient boosting algorithm that handles categorical features natively without requiring extensive preprocessing [43]. The objective function follows the standard boosting structure but incorporates a unique mechanism for processing categorical features:

(15) $$L_{CatBoost}\left(\theta \right)=\sum_{i=1}^{n}l\left(\hat{y_{i}},y_{i}\right)+\gamma \sum_{j=1}^{d}R_{j}\left(\theta \right)$$

where $R_{j}\left(\theta \right)$ represents the regularization term for categorical features. Hyperparameters such as the depth of the trees, the learning rate, and the number of boosting iterations are optimized using Optuna to minimize the loss:

(16) $$\theta^{*}=argmin_{\theta }L_{CatBoost}\left(\theta \right)$$

### 3.5. Classification Using Deep Neural Network Model

The DNN utilized for the classification of histopathological images is designed with a fully connected feed-forward architecture, chosen for its balance between computational efficiency and strong performance in image-based medical diagnostics. The network is composed of three dense layers, optimized to extract meaningful patterns from the hybrid feature set obtained from the MobileNetV2 and EfficientNetB3 models.

The DNN is initialized with a “He Normal” weight initializer, which helps maintain gradients during backpropagation, improving convergence. The hidden layers use the Exponential Linear Unit (ELU) activation function to enhance learning dynamics and avoid vanishing gradients. The final layer employs a softmax activation function to perform the multi-class classification task, mapping the output to a probability distribution across the five histopathological classes [34].

To prevent overfitting and improve generalization, the model employs early stopping with a patience of 10 epochs, ensuring that the best-performing model is retained. The Adamax optimizer was selected due to its computational efficiency and robustness for handling sparse gradients. Table 2 summarizes the architecture of the DNN model used in the classification task.

The network can be mathematically described as follows; let the input feature vector *X* be passed through the dense layers:

(17) $$Z_{1}=\mathrm{ELU}\left(W_{1}X+b_{1}\right)$$

(18) $$Z_{2}=\mathrm{ELU}\left(W_{2}Z_{1}+b_{2}\right)$$

(19) $$\hat{Y}=\mathrm{Softmax}\left(W_{3}Z_{2}+b_{3}\right)$$

where $Z_{1}$, $Z_{2}$ are the outputs of the hidden layers, $W_{1}$, $W_{2}$, $W_{3}$ are the weight matrices, $b_{1}$, $b_{2}$, $b_{3}$ are the bias terms and $\hat{Y}$ is the predicted output after applying the SoftMax function. The loss function used to optimize the network is sparse categorical cross-entropy, defined as:

(20) $$L\left(Y,\hat{Y}\right)=-\sum_{i=1}^{n}Y_{i}\log\left(\hat{Y_{i}}\right)$$

where Y represents the true labels and $\hat{Y}$ is the predicted probability for each class. The architecture of this DNN was selected for its ability to handle the large feature set extracted by MobileNetV2 and EfficientNetB3. The model strikes an optimal balance between high classification accuracy and computational efficiency, with fewer than 13 million parameters. This makes the model particularly suitable for deployment in real-time medical diagnostics, especially in resource-constrained environments such as point-of-care devices or embedded systems used by pathologists [35]. Finally, the model’s hyperparameters were optimized using Optuna, leveraging 5-fold cross-validation to ensure robustness and prevent overfitting.

## 4. Results and Discussion

**Figure 3:**
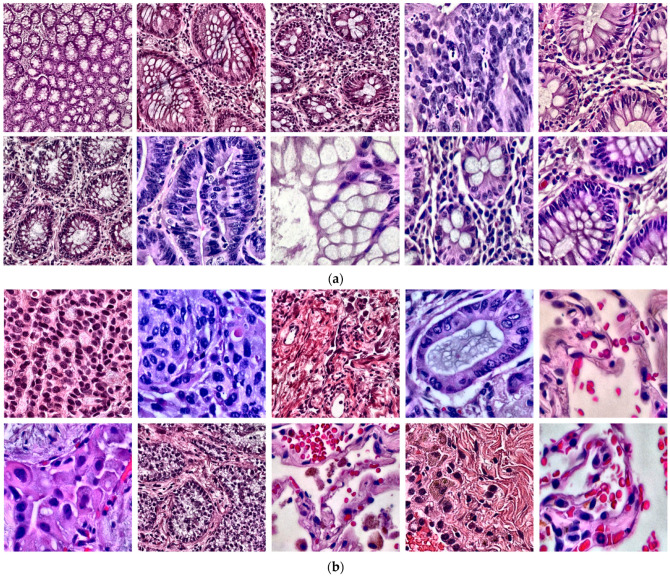
Sample histopathological images of cancer tissues: (**a**) Enhanced images of colon adenocarcinoma and colon benign tissue using CLAHE; (**b**) Enhanced images of lung adenocarcinoma, lung squamous cell carcinoma, and benign lung tissue using CLAHE.

The proposed MEGWO-LCCHC model was implemented using Python version 3.14 on a PC with an i9-13900KF processor, 3 GHz clock speed, 64 GB of RAM, NVIDIA RTX A4500 with 20 GB of memory, and a 1 TB PCIe HDD. Figure 3 illustrates a sample image of colon and lung tissues from the dataset used in this study. The MEGWO-LCCHC method was applied to each ML algorithm as well as the lightweight DNN model.

In Section 4.1, the results are analyzed, focusing on the performance metrics of the models. Section 4.2 delves into the limitations of this study, comparing the findings with the methods discussed in the literature review, and outlining considerations for future experiments.

### 4.1. Result Analysis

**Figure 4:**
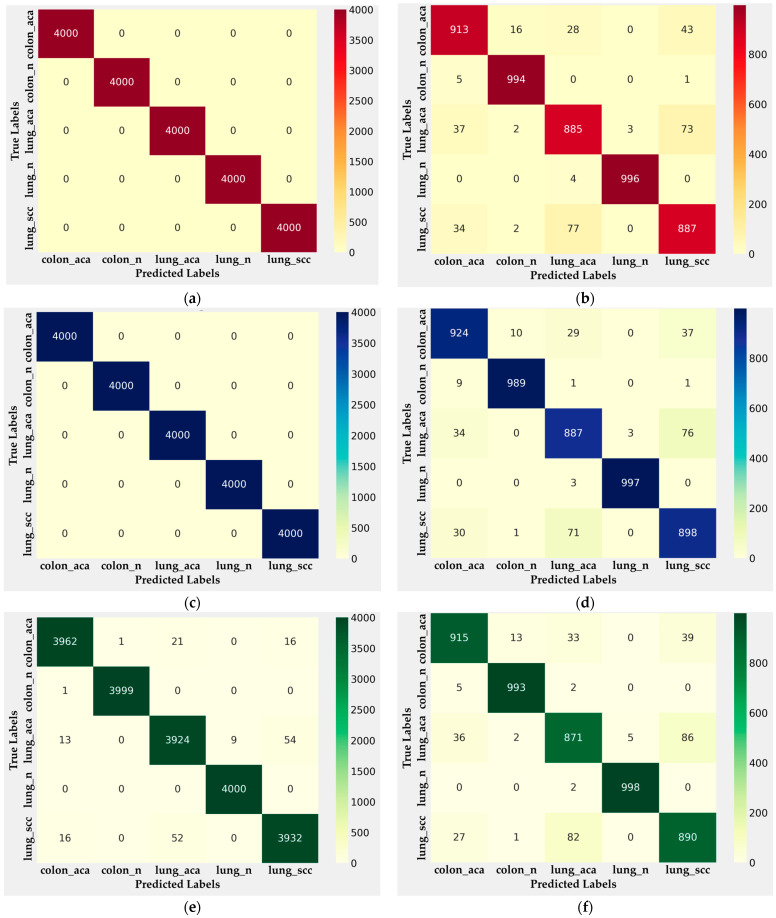

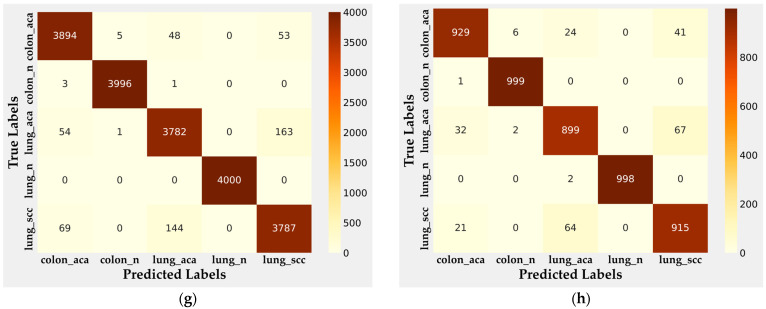
Confusion matrices illustrating the classification performance of the MEGWO-LCCHC approach across different classifiers: (**a**) XGBoost during Training; (**b**) XGBoost during Testing; (**c**) LightGBM during Training; (**d**) LightGBM during Testing; (**e**) CatBoost during Training; (**f**) CatBoost during Testing; (**g**) Lightweight DNN during Training; (**h**) Lightweight DNN during Testing.

The performance metrics of the proposed MEGWO-LCCHC method were evaluated using multiple classifiers during both the training and testing phases, following an 80:20 train-test split, as supported by previous studies that recommend this ratio for optimal model performance [15,16,17,18,19,20]. Figure 4 presents the confusion matrices generated during the classification of colon and lung cancer tissues by the XGBoost, LightGBM, CatBoost, and Lightweight DNN classifiers.

The confusion matrices from the training phase, particularly for XGBoost and LightGBM in Figure 4a,c, demonstrate strong classification capabilities, with nearly perfect predictions of benign and malignant samples across multiple classes. However, minor misclassifications, notably in the lung adenocarcinoma and squamous cell carcinoma categories, were observed, indicating areas for further refinement. This is further confirmed by the results from the testing phase (Figure 4b,d), which reveal slightly higher rates of misclassification in these classes, particularly with unseen data.

In contrast, the CatBoost classifier’s performance, as seen in Figure 4e,f, shows a high degree of accuracy across both phases, with the model maintaining robust classification performance. The model’s high true positive rates across all classes, particularly in the lung benign and colon adenocarcinoma categories, reflect its ability to generalize well to new data. Similarly, the Lightweight DNN classifier demonstrated strong performance in Figure 4g,h, maintaining a lower rate of misclassification, particularly in the lung cancer categories, while showcasing relatively high precision and recall values.

**Figure 5:**
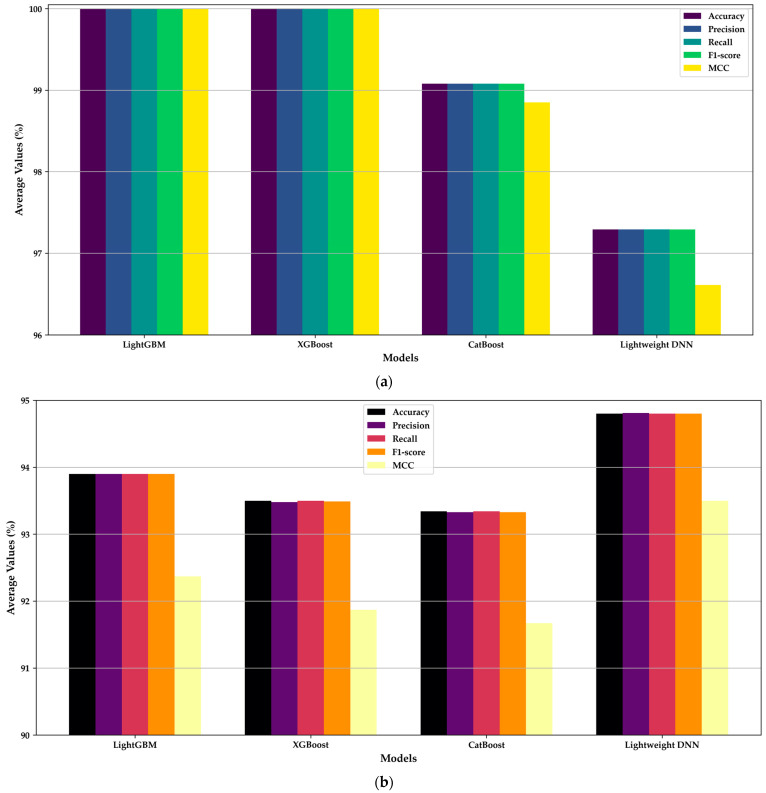
Average evaluation metrics of the MEGWO-LCCHC method across the 80:20 training and testing phases for all classifiers: (**a**) Metrics during the training phase; (**b**) Metrics during the testing phase.

Table 3 and Figure 5a highlight the average evaluation metrics for the MEGWO-LCCHC method during the training phase. Both LightGBM and XGBoost achieved perfect scores (100%) across all metrics, including accuracy, precision, recall, F1-score and Matthews Correlation Coefficient (MCC). While such performance is impressive, it raises concerns about potential overfitting, suggesting that these models may have learned specific nuances and noise from the training data rather than focusing on general patterns. CatBoost, on the other hand, achieved a near-perfect accuracy of 99.09%, with balanced metrics across precision, recall, and F1-score, indicating a more cautious learning approach that mitigates overfitting risks. The Lightweight DNN model also demonstrated strong performance with an accuracy of 97.29%, effectively balancing computational efficiency and predictive power.

During the testing phase, as shown in Table 4 and Figure 5b, the evaluation metrics showed a decline for LightGBM and XGBoost, with accuracy dropping to 93.9% and 93.5%, respectively. This contrast reflects a diminished generalization capability, as the models encountered new, unseen data. In contrast, CatBoost maintained strong performance during testing, with an accuracy of 93.34%, highlighting its robustness and capacity for generalization. While LightGBM and XGBoost slightly outperformed CatBoost in the testing phase alone, CatBoost’s balanced metrics across both phases reinforce its reliability and make it a suitable choice for datasets that require consistent performance across varied samples, such as those with multiple cancer tissue types. Interestingly, the Lightweight DNN model outperformed the other classifiers during testing, achieving an accuracy of 94.8%. This underscores the importance of model optimization and architecture design, as the DNN’s balanced computational efficiency and predictive power make it particularly well-suited for real-time medical applications.

**Figure 6:**
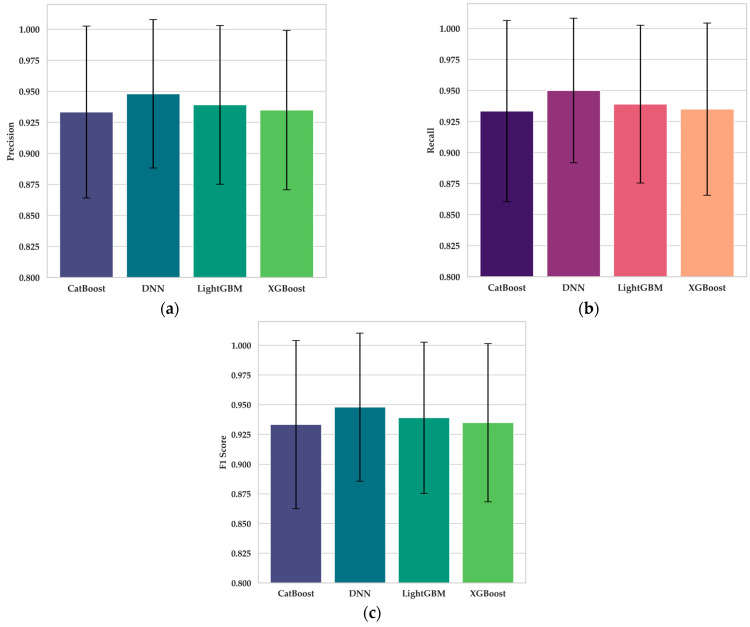
Confidence interval analysis of testing classification metrics for the MEGWO-LCCHC system in colon and lung cancer detection models: (**a**) Precision; (**b**) Recall; (**c**) F1-score.

The quantitative analysis of classifier performance metrics, specifically precision, recall, and F1-score, highlights the robust diagnostic capabilities of all evaluated models: DNN, XGBoost, LightGBM, and CatBoost. Figure 6a, which presents precision values, shows minimal variability across models. The DNN model achieved a mean precision of 0.948 (95% CI: 0.888–1.008), followed closely by LightGBM (0.939, 95% CI: 0.875–1.003), XGBoost (0.935, 95% CI: 0.871–0.999), and CatBoost (0.933, 95% CI: 0.864–1.003). The ANOVA test for Precision (*p* = 0.969) confirms that these differences are not statistically significant, indicating comparable effectiveness among models in accurately identifying cancerous and non-cancerous tissue samples.

The recall analysis, depicted in Figure 6b, reveals slightly higher variability among models, which is particularly relevant for distinguishing between benign and malignant tissue types. The DNN model again showed the highest mean Recall (0.950, 95% CI: 0.892–1.008), followed by LightGBM (0.939, 95% CI: 0.875–1.003), XGBoost (0.935, 95% CI: 0.866–1.004), and CatBoost (0.933, 95% CI: 0.860–1.006). The Kruskal-Wallis test for Recall (*p* = 0.586) indicates that, while some variability exists, it is not statistically significant, suggesting that all models exhibit consistent sensitivity across different cancer tissue types.

For F1-score, which balances precision and recall, all models maintained high and comparable values, as shown in Figure 6c. The DNN model marginally outperformed others, with a mean F1-score of 0.948 (95% CI: 0.886–1.010), followed by LightGBM (0.939, 95% CI: 0.875–1.003), XGBoost (0.935, 95% CI: 0.868–1.001), and CatBoost (0.933, 95% CI: 0.863–1.004). The ANOVA test for F1-score (*p* = 0.972) further supports that these models are statistically indistinguishable in their balanced diagnostic performance.

The confidence intervals (CIs) in Figure 6a–c provide additional insights into model consistency. Instances where CIs slightly exceed 1.0 are due to computational rounding in error estimation and do not imply diagnostic probabilities beyond theoretical limits. Instead, these intervals underscore each model’s near-optimal performance, with the DNN’s computational efficiency and balanced diagnostic accuracy suggesting its suitability for high-throughput, real-time clinical applications.

The classification performance of XGBoost, LightGBM, CatBoost, and Lightweight DNN was comprehensively evaluated across multiple cancer tissue types, with detailed metrics presented in Table 5, Table 6, Table 7 and Table 8. During the training phase, both XGBoost and LightGBM achieved perfect metrics (100%) across all evaluation categories, including accuracy, precision, recall, F1-score, and MCC. While such results indicate strong learning from the training data, they also suggest a risk of overfitting; In contrast, CatBoost achieved a commendable average accuracy of 99.08%, coupled with balanced metrics across all categories, reflecting effective learning with a reduced tendency toward overfitting. The Lightweight DNN model demonstrated an accuracy of 97.29%, balancing computational efficiency and predictive performance, making it particularly suitable for real-time applications where processing speed is critical.

In the testing phase, XGBoost and LightGBM exhibited a decline in metrics, with accuracies reducing to 93.5% and 93.9%, respectively. This drop highlights their diminished generalization capabilities when applied to new, unseen data, particularly for malignant tissue types such as lung_aca and lung_scc. In these categories, both models demonstrated reduced precision and recall, which may impact their reliability in clinical applications where accurate classification of malignant samples is paramount. In contrast, CatBoost maintained robust performance during testing, with an accuracy of 93.34%, demonstrating resilience across both benign and malignant tissue types. Although LightGBM and XGBoost marginally outperformed CatBoost on certain testing metrics, CatBoost’s consistency across both training and testing phases reinforces its reliability, particularly in datasets with complex class distributions and multiple cancer tissue types. Notably, the Lightweight DNN model achieved the highest testing accuracy at 94.8%, outperforming other classifiers in terms of generalization. This superior performance underscores the importance of model architecture in achieving a balance between computational efficiency and predictive power, positioning the DNN model as particularly advantageous for high-throughput, real-time diagnostic applications.

In benign tissue classes, such as colon_n and lung_n, all models exhibited high precision, recall, and F1-scores across both training and testing phases. This strong performance can be attributed to the dataset’s balance in these categories, as no additional images from the National Cancer Institute’s GDC Data Portal were introduced, thereby minimizing variability in these classes.

Conversely, malignant classes such as lung_aca and lung_scc posed a greater challenge, with all models recording comparatively lower performance metrics in these categories during testing. XGBoost and LightGBM demonstrated notable declines in these classes, with XGBoost attaining precision and recall values of 89.03% and 88.5%, respectively, for lung_aca, and LightGBM achieving 89.51% and 88.7% for the same class. CatBoost exhibited more stable results in these challenging classes, with precision and recall ranging between 87.98% and 89% for lung_aca and lung_scc. The Lightweight DNN model showed a slightly superior capacity to generalize in these malignant tissue classes, attaining precision and recall values between 89% and 91% for lung_aca and lung_scc, suggesting enhanced robustness in challenging classification scenarios.

Table 9 provides a detailed comparison of the false positive (FP) and false negative (FN) rates across five cancer classes using the MEGWO-LCCHC method with different classifiers: LightGBM, XGBoost, CatBoost, and the lightweight DNN. The FP and FN rates are critical metrics for evaluating the clinical applicability of diagnostic models, as they reflect the likelihood of misclassifications that could lead to under- or overdiagnosis.

The lightweight DNN consistently achieved the lowest FN rates across all cancer classes, with particularly strong performance in benign tissue classification for both colon and lung cancers. For instance, the FN rate for lung_n was only 0.2%, and no false positives were recorded. This demonstrates the DNN’s ability to accurately distinguish between malignant and benign tissues, a crucial requirement for early-phase diagnostics.

CatBoost showed competitive performance, particularly in minimizing FP rates for colon_aca (1.7%) and colon_n (0.125%). However, it exhibited slightly higher FN rates for lung_ssc (12.9%) and lung_aca (12.0%), indicating a tendency to underdiagnose malignant cases. LightGBM and XGBoost performed comparably, with FN rates ranging from 10.1% to 11.5% for lung_aca and lung_ssc. While these models demonstrated lower FP rates for benign tissues, their FN rates for malignant cases suggest the need for further optimization to improve sensitivity, especially in high-risk categories like lung cancers. In general, the DNN exhibited the best balance between FP and FN rates, with its FN rates significantly lower across all classes compared to the other classifiers. This reinforces its suitability for real-time clinical applications, where minimizing both false positives and false negatives is paramount for accurate and timely diagnosis.

**Figure 7:**
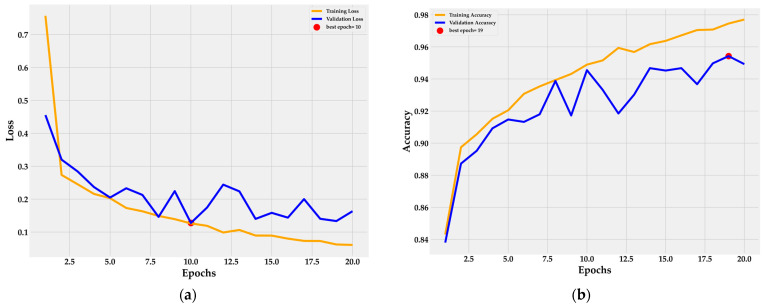
Loss and accuracy curves for the MEGWO-LCCHC method with the lightweight DNN: (**a**) Loss curve; (**b**) Accuracy curve.

The training and validation loss curves in Figure 7a demonstrate the model’s convergence after 20 epochs, with early stopping identifying the optimal epoch at 10, effectively preventing overfitting. The continuous decline in training loss indicates strong learning from the training data, while the stabilization of validation loss beyond epoch 10 reflects the model’s ability to generalize well to unseen data.

Figure 7b highlights the model’s training accuracy nearing 98%, showcasing its efficiency in capturing data patterns. Validation accuracy stabilizes around 95%, reaching optimal performance at epoch 19. Despite minor fluctuations in the validation curve, the model maintains strong adaptability to validation data without overfitting. This balance between training and validation metrics underscores the effectiveness of the MEGWO-LCCHC method in classifying lung and colon cancer tissues, maintaining high generalization across both phases. The findings further affirm the robustness of the MEGWO-LCCHC method in maintaining balanced performance during both training and testing. The integration of early stopping and the GWO for feature reduction not only enhances training efficiency but also strengthens model resilience. These aspects make the proposed method highly suitable for large-scale classification tasks involving complex histopathological images, ensuring both accuracy and generalization in clinical applications.

**Figure 8:**
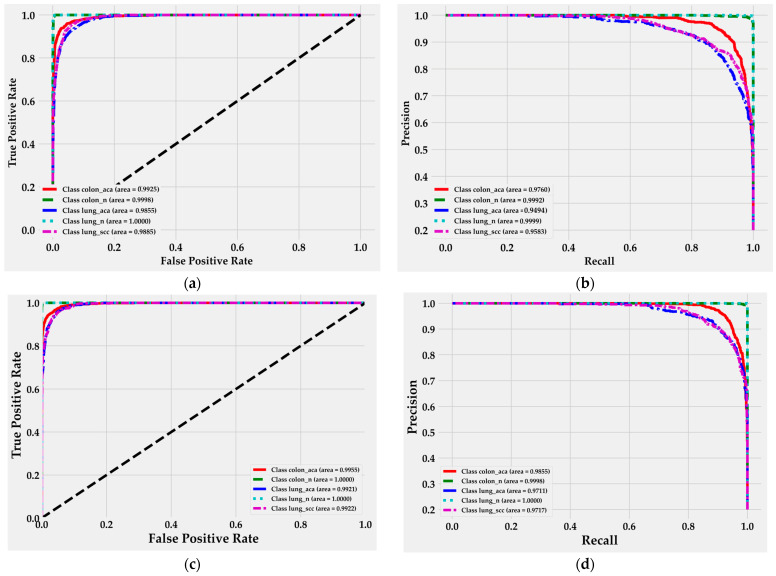
ROC and PR curve analysis of the MEGWO-LCCHC system using an 80:20 training/testing split: (**a**) ROC curve for CatBoost in the testing phase; (**b**) PR curve for CatBoost in the testing phase; (**c**) ROC curve for the Lightweight DNN in the testing phase; (**d**) PR curve for the Lightweight DNN in the testing phase.

The ROC and Precision-Recall (PR) curves presented in Figure 8a,b offer a deeper understanding of the classification performance of the MEGWO-LCCHC method using both CatBoost and Lightweight DNN models. The ROC curves (Figure 8a) illustrate the balance between the true positive rate (sensitivity) and false positive rate, while the PR curves (Figure 8b) highlight the trade-off between precision and recall, particularly for imbalanced classes such as malignant tissues.

For the CatBoost classifier, the ROC curves demonstrate near-perfect performance in distinguishing between benign tissue classes, with lung_n achieving an AUC of 1.0 and colon_n reaching 0.9925. The colon_aca class also shows a high AUC value of 0.9925, confirming its strong ability to classify colon adenocarcinoma. However, the performance slightly decreases for malignant lung tissue classes (lung_aca and lung_scc), which yield AUC values of 98% or higher, indicating robust classification but with potential for further improvement.

Similarly, the PR curves show a high level of performance for benign tissue classes, with CatBoost achieving values close to 100%. However, for malignant lung tissues (lung_aca and lung_scc), the AUC values drop to 94.94% and 95.83%, respectively. These results suggest that, while the classifier performs strongly across most classes, there is room for refinement in the classification of malignant tissues.

For the Lightweight DNN model, the ROC curves in the testing phase (Figure 8c) exhibit consistently strong performance across all tissue classes, with AUC values above 99%. Malignant lung tissues (lung_aca and lung_scc) perform well, achieving AUC values of 0.9921 and 0.9922, while benign tissues maintain near-perfect performance. The PR curves (Figure 8d) reflect similar trends, with the colon_aca class reaching an AUC of 98.55%, and malignant lung tissues achieving AUC values around 97%.

This analysis reinforces the strength of the MEGWO-LCCHC method, particularly in balancing precision and recall across different tissue types, though further improvements could be achieved with a more generalized dataset, especially in malignant lung tissue classification.

### 4.2. Discussion

The results presented in this study emphasize the advantages and challenges of the MEGWO-LCCHC method in the classification of lung and colon cancer tissue. Despite the advances in feature extraction and image enhancement techniques like CLAHE, which capture essential spatial features in histopathological images, the literature emphasizes the importance of hyperparameter optimization to guarantee the best performance in both DL and ML models [18,23,24,26,27,32,44]. Feature reduction further ensures better outcomes in terms of precision and F1-score, crucial metrics for evaluating models intended for clinical applications. The present study confirms the importance of contrast enhancement, especially in a generalized dataset that includes malignant tissues.

The MEGWO-LCCHC method demonstrated robust performance across various classifiers. However, ongoing efforts to improve generalizability and mitigate overfitting are essential for real-world clinical applications [10,11,12,13]. Future studies should explore alternative architectures and additional feature engineering techniques to enhance diagnostic accuracy [34]. Given the inherent variability in real-world data, it’s important to acknowledge that the LC25000 dataset was augmented from just 750 original images to 25,000. While augmentation is a valuable technique for increasing dataset size and improving model robustness, it doesn’t introduce real variability in the data [18,22,23,24,33]. This limitation became apparent, as the dataset from the GDC Cancer Data Portal included only malignant tissue images. Despite some discrepancies between training and testing phases, the models captured the classification of five cancer classes effectively, although exceptions occurred, especially with the malignant tissue classes, due to this imbalance.

The advantages of the MEGWO-LCCHC model lie in its feature extraction approach, which combines MobileNetV2 and EfficientNetB3—two models that have demonstrated robustness in similar applications. The use of feature reduction with GWO accelerates training times and enhances computational efficiency. This lightweight approach has been sought in previous research, focusing on models that can be deployed in complex tasks like multi-class cancer identification [34,35].

CatBoost demonstrated superior generalization, particularly in the classification of benign tissue, achieving near-perfect precision and recall scores. The model’s robustness against overfitting is noteworthy, especially given the imbalanced dataset, where malignant tissue images dominated. LightGBM and XGBoost excelled during the training phase, achieving perfect metrics, but exhibited slight declines in performance during testing, highlighting areas for potential improvement in generalization. This drop in testing performance suggests that while these gradient-boosting models learned patterns effectively from the training data, further tuning is required to improve their performance on unseen data. While ensemble learning methods, such as those utilizing ML models, can be effective, the goal of this research is to develop a framework that captures features effectively without the need for excessive model combinations [30,44]. Furthermore, deploying AI models in clinical environments often requires specialized hardware, such as GPUs or TPUs, which may not be readily available. Thus, our lightweight approach remains highly relevant [18,22,33].

Conversely, CatBoost and DNN exhibited strong generalization capabilities, with no significant signs of overfitting, making them reliable for real-time clinical applications. CatBoost particularly stood out for its ability to balance precision and generalization. In contrast, the lightweight DNN showed an excellent balance between performance and computational efficiency. This architecture ensures that it is well-suited for real-time clinical applications [35].

The limitations of existing methodologies in lung and colon cancer detection are evident in their limited capacity to generalize across diverse clinical settings. Many current approaches depend on augmented datasets, which, while valuable for increasing dataset size, do not introduce genuine variability and may lead to overfitting on synthetic patterns rather than real-world data nuances [22,23,24,25,26,27,30]. Moreover, the high computational demands of advanced model architectures restrict their deployment in clinical settings, where rapid, real-time diagnostics are critical. The MEGWO-LCCHC method addresses some of these gaps by incorporating new histopathological images from the National Cancer Institute GDC Data Portal, enhancing data diversity and improving generalizability. Additionally, our lightweight model architecture is designed to balance computational efficiency with accuracy, making it more suitable for practical, real-time diagnostic applications.

Table 10 presents a comparative analysis of the MEGWO-LCCHC method alongside other state-of-the-art approaches that have been applied to the LC25000 dataset. Notably, the MEGWO-LCCHC method includes an extended dataset integrating histopathological images from the National Cancer Institute GDC Data Portal, unlike the other methods that rely solely on the augmented LC25000 dataset. While these models demonstrate high accuracy (≥99%) across all metrics, it is essential to highlight the inherent limitations in their evaluation. Metrics derived from augmented datasets often fail to capture the variability and complexity of real-world clinical scenarios, potentially inflating performance metrics due to synthetic data patterns.

The MEGWO-LCCHC method’s testing accuracy of 94.8% with the lightweight DNN, while slightly lower than the near-perfect results reported by methods applied solely to LC25000, offers a more realistic benchmark for clinical applicability. This discrepancy underscores the value of incorporating real histopathological images, which introduce natural variability and better simulate real-world conditions. In addition, this framework prioritizes lightweight architecture and computational efficiency, addressing critical challenges for deploying AI solutions in clinical settings, particularly in resource-limited environments.

The limitations of current methodologies underscore the challenges of generalizing models across diverse clinical settings. The heavy reliance on augmented datasets often restricts the ability of these approaches to generalize effectively across varying patient populations and tissue heterogeneity. Accurate diagnosis of dysplastic and early-phase lesions is critical, as early intervention can substantially improve survival rates while reducing the need for aggressive treatments. The MEGWO-LCCHC framework, with its focus on lightweight and computationally efficient models, demonstrates strong potential for real-time clinical applications, particularly in early-phase diagnostics where precision and timeliness are crucial. By automating the analysis of histopathological images, this AI-based tool assists pathologists by identifying regions that may need further review, reducing manual workload and minimizing the risk of oversight in subtle cases. Importantly, the tool supports diagnostic consistency and reliability, especially in challenging cases, while still relying on the expertise of medical professionals to finalize reports. This framework enhances workflow efficiency, allowing pathologists to concentrate on complex cases, and is designed to generalize beyond benchmark datasets to address the inherent variability of real-world clinical pathology [22,23,24,25,26,27,28,29,30,34,44].

Future directions should focus on the acquisition of larger and more diverse datasets of histopathological images to enhance model generalization further. Additionally, incorporating multimodal data—such as patient medical records, laboratory results, and treatment histories—could significantly improve the diagnostic capabilities of ML models like CatBoost, LightGBM, and XGBoost. Expanding the framework to include such data would allow these models to better generalize across patient populations and cancer types, enabling their adoption in diverse clinical settings [18,22,23,24,33,35].

## 5. Conclusions

In this study, we proposed the MEGWO-LCCHC method for the classification of lung and colon cancer using histopathological images. The integration of MobileNetV2 and EfficientNetB3 with the GWO for feature extraction and reduction provided a lightweight and computationally efficient approach, suited for real-time clinical applications. The inclusion of additional histopathological images from the National Cancer Institute GDC Data Portal further improved the model’s generalizability by replacing augmented data with real samples, particularly enhancing classification performance for malignant tissues.

The findings demonstrate that the lightweight DNN achieved the highest accuracy of 94.8% during testing, outperforming other models. CatBoost demonstrated robust generalization, particularly for benign tissue classification, while LightGBM and XGBoost achieved high training metrics but exhibited performance declines during testing, underscoring the need for further optimization to improve their generalization capabilities. Table 10 highlights the relevance of incorporating real-world data to provide a more accurate benchmark compared to methods relying solely on augmented datasets.

Future work aims to establish collaborations with clinical laboratories and pathologists to enhance the dataset through expert validation and curation. This initiative seeks to ensure that the enhanced images adhere to the highest diagnostic standards and accurately represent real-world clinical scenarios. Focus will be given to early-phase and dysplastic lesions, which present significant diagnostic challenges. Incorporating expert-reviewed data is expected to enhance the clinical applicability and generalizability of the framework, particularly in resource-limited settings. Moreover, integrating multimodal data sources—such as clinical records, treatment histories, genomic information, and laboratory findings—holds substantial potential to complement histopathological patterns, thereby improving diagnostic accuracy and model robustness [18,22,23,24,32,33,35].

This approach could substantially enhance the robustness and generalizability of models across diverse patient populations. Additionally, exploring innovative feature extraction techniques and alternative architectures could further refine the diagnostic potential of the MEGWO-LCCHC framework. Tailored specifically for early-phase lesion detection, this framework has the capacity to improve sensitivity and specificity in these challenging scenarios, ensuring its adaptability and reliability in real-world clinical environments [34].

## Figures and Tables

**Table 1:** Details of the dataset LC25000 mixed with new samples from National Cancer Institute GDC Data Portal.

Class Name	Dataset Used	Description	No. of Samples
Colon_aca	LC25000 + National Cancer Institute GDC Data Portal	Colon Adenocarcinoma	5000
Colon_n	LC25000	Colon Benign Tissue	4000 + 1000
Lung_aca	LC25000 + National Cancer Institute GDC Data Portal	Lung Adenocarcinoma	4000 + 1000
Lung_n	LC25000	Lung Benign Tissue	5000
Lung_ssc	LC25000 + National Cancer Institute GDC Data Portal	Lung Squamous Cell Carcinoma	4000 + 1000
	Total Number of Samples		25,000

**Table 2:** Deep neural network architecture for histopathology classification.

Layer	Type	Units	Activation	Kernel Initializer
1	Dense	1000	ELU	He Normal
2	Dense	500	ELU	He Normal
3	Dense	5	Softmax	-

**Table 3:** Average evaluation metrics for the training phase (80%) of the MEGWO-LCCHC method with different classifiers.

Model	Accuracy	Precision	Recall	F1-Score	MCC
LightGBM	100	100	100	100	100
XGBoost	100	100	100	100	100
CatBoost	99.08	99.08	99.08	99.08	98.85
Lightweight DNN	97.29	97.29	97.29	97.29	96.61

**Table 4:** Average evaluation metrics for the testing phase (20%) of the MEGWO-LCCHC method with different classifiers.

Model	Accuracy	Precision	Recall	F1-Score	MCC
LightGBM	93.9	93.9	93.9	93.9	92.37
XGBoost	93.5	93.48	93.5	93.49	91.87
CatBoost	93.34	93.33	93.34	93.33	91.67
Lightweight DNN	94.8	94.81	94.8	94.8	93.5

**Table 5:** Classification report for the training and testing phase of the MEGWO-LCCHC method with XGBoost.

Class	Precision	Recall	F1-Score
	Training Phase (80%)		
Colon_aca	100	100	100
Colon_n	100	100	100
Lung_aca	100	100	100
Lung_n	100	100	100
Lung_ssc	100	100	100
Average	100	100	100
	Testing Phase (20%)		
Colon_aca	92.32	91.30	91.80
Colon_n	98.03	99.40	98.71
Lung_aca	89.03	88.50	88.77
Lung_n	99.70	99.60	99.65
Lung_ssc	88.35	88.70	88.52
Average	93.48	93.34	93.49

**Table 6:** Classification report for the training and testing phase of the MEGWO-LCCHC method with LightGBM.

Class	Precision	Recall	F1-Score
	Training Phase (80%)		
Colon_aca	100	100	100
Colon_n	100	100	100
Lung_aca	100	100	100
Lung_n	100	100	100
Lung_ssc	100	100	100
Average	100	100	100
	Testing Phase (20%)		
Colon_aca	92.68	92.40	92.54
Colon_n	98.90	98.90	98.90
Lung_aca	89.51	88.70	89.10
Lung_n	99.70	99.70	99.70
Lung_ssc	88.74	89.80	89.26
Average	93.90	93.90	93.90

**Table 7:** Classification report for the training and testing phase of the MEGWO-LCCHC method with CatBoost.

Class	Precision	Recall	F1-Score
	Training Phase (80%)		
Colon_aca	99.25	99.05	99.15
Colon_n	99.98	99.98	99.98
Lung_aca	98.17	98.10	98.14
Lung_n	99.78	100	99.89
Lung_ssc	98.25	98.30	98.28
Average	99.08	99.09	99.08
	Testing Phase (20%)		
Colon_aca	93.08	91.50	92.28
Colon_n	98.41	99.30	98.86
Lung_aca	87.98	87.10	87.54
Lung_n	99.50	99.80	99.65
Lung_ssc	87.68	89.00	88.34
Average	93.33	93.34	93.33

**Table 8:** Classification report for the training and testing phase of the MEGWO-LCCHC method with lightweight DNN.

Class	Precision	Recall	F1-Score
	Training Phase (80%)		
Colon_aca	96.86	97.35	97.10
Colon_n	99.85	99.90	99.87
Lung_aca	95.14	94.55	94.84
Lung_n	100	100	100
Lung_ssc	94.60	94.67	94.63
Average	97.29	97.29	97.29
	Testing Phase (20%)		
Colon_aca	94.50	92.90	93.69
Colon_n	99.20	99.90	99.55
Lung_aca	90.89	89.90	90.39
Lung_n	100	99.80	99.89
Lung_ssc	89.44	91.50	90.45
Average	94.81	94.8	94.8

**Table 9:** False positive and false negative rates for the MEGWO-LCCHC method across different classifiers during the testing phase.

Model/Test Set	Colon_aca		Colon_n		Lung_aca		Lung_n		Lung_ssc	
	FP (%)	FN (%)	FP (%)	FN (%)	FP (%)	FN (%)	FP (%)	FN (%)	FP (%)	FN (%)
LightGBM	1.825	7.6	0.275	1.1	2.6	11.3	0.075	0.3	2.85	10.2
XGBoost	1.9	8.7	0.5	0.6	2.725	11.5	0.075	0.4	2.925	11.3
CatBoost	1.7	8.5	0.4	0.7	2.975	12.9	0.125	0.2	3.125	11.0
DNN	1.35	7.1	0.2	0.1	2.25	10.1	0	0.2	2.7	8.5

**Table 10:** Comparative MEGWO-LCCHC method with recent algorithms.

Methods	Dataset Used	Accuracy	Precision	Recall	F1-Score
MPADL-LC3 [24]	LC25000	99.27	98.18	98.17	98.17
Custom CNN + PCA, FWHT, DWT [22]	LC25000	99.60	99.60	99.90	99.60
IWSACAE-LCCD [18]	LC25000	99.41	98.52	98.51	98.51
BERTL-HIALCCD [23]	LC25000	99.22	98.07	98.06	98.06
BICLCD-TSADL [32]	LC25000	99.33	98.31	98.31	98.31
EfficientNetV2-L [33]	LC25000	99.97	99.90	99.97	99.97
LW-MS-CCN with XAI [35]	LC25000	99.20	99.16	99.36	99.16
HIELCC-EDL [44]	LC25000	99.6	99.0	99.0	99.0
MEGWO-LCCHC with DNN	LC25000 + National Cancer Institute GDC Data Portal	94.8	94.81	94.8	94.8

## Data Availability

The datasets used in this study are publicly available. The enhanced LC25000 dataset, including additional images and CLAHE preprocessing, can be accessed via the following links: https://github.com/AlbertoGudinoOchoa/Enhanced-LC25000-CLAHE-Dataset-Cancer-Classification (accessed on 14 October 2024). and https://drive.google.com/drive/folders/1aQNez61naAiuveaQlSzJ2VBsMI5_KUYm (accessed on 14 October 2024). The dataset is distributed under a Creative Commons Attribution-ShareAlike 4.0 International (CC BY-SA 4.0) license, which allows sharing, adaptation, and redistribution of the material, provided appropriate credit is given and any derivative works are shared under the same license. Full details of the license can be found at Creative Commons License.
